# Cross-Cultural Adaptation of the Social Vulnerability Index for Use in the Dutch Context

**DOI:** 10.3390/ijerph14111387

**Published:** 2017-11-14

**Authors:** Steven Bunt, Nardi Steverink, Melissa K. Andrew, Cees P. van der Schans, Hans Hobbelen

**Affiliations:** 1 Research Group in Healthy Ageing, Allied Health Care and Nursing, Hanze University Groningen, University of Applied Sciences, 9714 CA Groningen, The Netherlands; c.p.van.der.schans@pl.hanze.nl (C.P.v.d.S.); j.s.m.hobbelen@pl.hanze.nl (H.H.); 2 Department of Sociology, Faculty of Behavioural and Social Sciences, University of Groningen, 9712 TG Groningen, The Netherlands; b.j.m.steverink@rug.nl; 3 Department of Health Psychology, University Medical Center Groningen, University of Groningen, 9713 AV Groningen, The Netherlands; 4 Department of Medicine (Geriatrics), Dalhousie University, Halifax, NS B3H 2E1, Canada; melissa.andrew@nshealth.ca; 5 Department of Rehabilitation, University Medical Center Groningen, University of Groningen, 9700 RB Groningen, The Netherlands; 6 Department of General Practice and Elderly Care Medicine, University of Groningen, University Medical Center Groningen, 9700 AD Groningen, The Netherlands

**Keywords:** social frailty, social vulnerability, frailty, index

## Abstract

Being able to identify socially frail older adults is essential for designing interventions and policy and for the prediction of health outcomes, both on the level of individual older adults and of the population. The aim of the present study was to adapt the Social Vulnerability Index (SVI) to the Dutch language and culture for those purposes. A systematic cross-cultural adaptation of the initial Social Vulnerability Index was performed following five steps: initial translation, synthesis of translations, back translation, a Delphi procedure, and a test for face validity and feasibility. The main result of this study is a face-valid 32 item Dutch version of the Social Vulnerability Index (SVI-D) that is feasible in health care and social care settings. The SVI-D is a useful index to measure social frailty in Dutch-language countries and offers a broad, holistic quantification of older people’s social circumstances related to the risk of adverse health outcomes.

## 1. Introduction

When people age, they are often confronted with (health) deficits and become increasingly frail and have increased risk of adverse health outcomes, institutionalization, and mortality. Frailty is a concept that describes a decreased reserve capacity and resistance to stressors causing vulnerability to adverse health outcomes. The concept of frailty has developed from a perspective that emphasizes physical aspects of frailty [1] to a more integral perspective that comprises the multidimensional aspects of frailty [2,3,4]. The underlying idea in these latter models is that frailty increases with the accumulation of physical, psychological, and social deficits (or problems), i.e., “deficit accumulation”. In this perspective Rockwood et al. acknowledge three domains: physical frailty, cognitive frailty, and social frailty [5]. Regarding the social domain, it is known that social conditions, such as social network, social participation, or social support, are related to health status and mortality of people [6,7]. Previously, we defined social frailty as a continuum of being at risk of losing, or having lost, social and general resources, activities, or abilities that are important for fulfilling one or more basic social needs during one’s life span [8]. Others have used the concept of social vulnerability to describe losses in the social domain that influence the ageing process and frailty of older adults [9], but there is no consensus yet on the question of whether social vulnerability is the same or different than social frailty. Therefore, we see both concepts as synonyms and will, in this paper, speak of social frailty.

While people age, different social resources, activities, and abilities affect the fulfilment of older adults’ social needs. These resources, activities, and abilities influence each other and are therefore important for both health and welfare care and policy-making. Therefore, identifying social frailty among older people is important. Since the concept of social frailty comprises a complex, dynamic interaction of resources, activities, and abilities for fulfilling the social needs of individual older adults, an index to measure social frailty could provide insights into their specific health- and social care needs. Although several Dutch multidimensional frailty instruments are available, in which limited aspects of social frailty are incorporated, to our knowledge, a specific Dutch instrument to measure social frailty is not available yet.

The Social Vulnerability Index (SVI) was developed for this purpose, providing a holistic quantification of social vulnerability among older people and appearing to be a valid measure [9,10,11]. The SVI is a multifactorial and multilevel index that consists of items that reflect particular aspects of a person’s social circumstances. Rather than focusing on one social dimension, the index (see Appendix A for a sample 40 item index) includes a broad range of social factors, thereby creating a holistic measure of social vulnerability. The factors to be considered have been compiled from population-based longitudinal studies in Canada and Europe. Factors that influence and describe an individual’s social circumstance, such as social support or social engagement (based on previous studies that suggested they were relevant), were selected in the original index. Factors that relate to socio-economic status (such as income adequacy or home ownership) were also included, as these factors are known for their potential to impact health status. In previous studies, it appeared to be a valid measure because it correlated with (yet was distinct from) frailty and predicted mortality and disability [9,10]. Importantly, the deficit accumulation approach does not specify that a certain number or specific list of items be included in the index, as long as a breadth of domains is included. This allows for flexibility, as the approach may be applied to existing datasets and adapted to different contexts as is being studied here. While the flexible deficit accumulation approach has proven useful and applicable in epidemiological studies and existing databases [9,10,11], developing a specific list of questions is a useful endeavour for application in clinical settings. The SVI potentially is a useful instrument, both on the population level for researchers (for example in epidemiological studies) and in clinical settings for health care and welfare professionals to measure social frailty in individual older adults.

In the SVI, a score of 0 is assigned if a social deficit is absent in an individual and 1 if it is present; intermediate values (0.5) are applied in cases of ordered response categories. For example, an individual scores 1 on the “lives alone” deficit if he/she reports living alone, and 0 if he/she does not. On the “do you ever feel you need more help” deficit, which has three response categories, possible scores are 0 if the answer is “never”, 0.5 for “sometimes”, and 1 for “often”. For each individual, the social vulnerability index generates a sum of the deficit scores, which is then divided by the number of social deficits considered (e.g., 40 in the example above) leading to a theoretical range of 0–1 when expressed as an index. Where it aids interpretation (for example, in regression models), the SVI may also be expressed in terms of the raw number of deficits (such that the rangs in the sample index would be 0–40. The conceptual idea is that an accumulation of social deficits, and therefore a higher score on the SVI, measures the degree of social frailty, which potentially leads to a risk of adverse (health) outcomes [9]. To our knowledge, there is no such instrument to measure social frailty in the Dutch language. As there are potential differences in culture influencing the applicability of such an index, cross-cultural adaptation is important. If measures are to be used across cultures, the items must not only be translated well linguistically, but also be adapted culturally in order to maintain the content validity of the instrument across different cultures. The term “cross-cultural adaptation” in this paper is used to describe a process that incorporates both language (translation) and cultural adaptation issues in the process of preparing the index for use in the Dutch context [12].

In order generate such an instrument, we undertook the cross-cultural adaptation of the original Social Vulnerability Index.

The goal of this paper is to adapt the Social Vulnerability Index for use in Dutch-speaking countries and to test its face validity and feasibility.

## 2. Materials and Methods

To adapt the Social Vulnerability Index (SVI) to the Dutch language and culture, we used guidelines for cross-cultural adaptation of measurement instruments [12] following five different steps. The Medical Ethical Committee of the University Medical Center Groningen evaluated the study and judged that it did not need ethical approval under Dutch law (approval code METc 2016/310).

Step 1 Initial translation

In the first step, we translated the 40 items of the original SVI. The original version of the SVI was translated twice independently into Dutch by two native Dutch persons both with a good understanding of English. All 40 items were regarded as potentially pertinent in this step.

Step 2 Synthesis of the translations

These two Dutch versions of the SVI were reviewed and discussed by a Dutch researcher (with a background in frailty and ageing) and the health care professional who performed one of the translations; differences in the two separate translations were discussed, and consensus was reached upon a synthesized version of the initial two translations.

Step 3 Back translation

A professional, native English language-speaking translator, without knowledge of concepts and purpose of the index, performed the back-translation. This back-translation was then compared to the initial index to screen for any relevant changes to the meaning of the items of the SVI made during the translation process. The comparison was performed by the two involved persons from the second step. Any relevant differences were discussed and reached consensus upon if necessary.

Step 4 Expert committee: A Delphi procedure

In the fourth step, an expert committee was constituted. Because we extended upon the minimal requirements of such a panel and involved a relative large group of experts, we applied a Delphi procedure, which is a technique to reach consensus on a particular issue [13]. In this procedure, a digital questionnaire was sent to a panel of experts. These experts were selected for their expertise and experience: they had to be social and/or medical scientist in the field of (social) frailty and ageing or health care- and social care professionals working with older people, and from a Dutch origin, preferably working in The Netherlands. These experts were considered to have relevant knowledge on the concept of social frailty, and on the applicability of items to measure this concept in the Dutch context. The expert panel was composed by contacting 20 experts by email to participate in the Delphi procedure. The scientific experts were selected based on their track record in their professional expertise, and the professional experts were selected based on their working experience and professional setting. A reminder was sent after three weeks if there was no response.

This Delphi procedure consisted of three rounds:

Round 1

In the first round, the panel of experts were asked to comment anonymously on the suitability of all items for use in the Dutch culture. They were asked to score every separate item of the Dutch version of the SVI (SVI-D) on a five-point Likert-scale (very unsuitable-unsuitable-neutral-suitable-very suitable) to judge the experiential and conceptual relevance of the item in the Dutch context, or in other words, to judge if they are relevant in the Dutch context. Next to that, there was the opportunity to give qualitative feedback on the clarity of language in the Dutch translation of the SVI on every item in order to judge the idiomatic and semantic meaning in the Dutch language of the separate items. Finally, there was a question whether the experts thought items were missing, and if so, which one(s).

Once all responses were collected, a new version of the questionnaire was designed based on the results of the first round for a second round in the Delphi-procedure. Only the items that were scored “suitable” or “very suitable” by 70% or more (cut-off point) of the expert panel in the first round were included in a new set of items for the second round. Also, the qualitative feedback on the clarity of language was processed in the separate items by the first author (SB), i.e., suggestions of the experts to change words and sentences were applied in the questionnaire.

Round 2

In the second round, the experts were asked to reconsider their initial opinion in light of the (anonymous) group results of the first round: they were asked to score “agree” or “not agree” on every separate item whether or not to include this item in the prefinal index. Again, there was the opportunity to give qualitative feedback on the clarity of language in the Dutch translation of the SVI on every item, to judge the idiomatic and semantic meaning in the Dutch language of the separate items. Once the responses were collected, only items that were agreed upon by 70% or more of the experts (cut-off point) were included in the pre-final version for the third round. In the literature, cut-offs between 55% and 100% are used [14]. The cut-off of 70% for consensus was chosen as a reasonable middle: if more than 70% of the experts agreed, items were included.

Round 3

In the third round, the panel of experts were asked one question: if they agreed (yes/no) upon the prefinal version of the SVI-D that was sent to them. Also, there was a possibility to express final comments on the SVI-D as a clarification of their answer. When present, these final comments of the experts are discussed in this paper.

Step 5 Testing the prefinal version: face validity and feasibility

In the fifth step of this cross-cultural adaptation process, face validity and feasibility of the instrument were examined. Two health care professionals administered the SVI-D in a sample of 28 community dwelling older people. These people were selected by both health care professionals from their current patient population in a convenience sample based on their willingness to participate. The purpose and procedure of the research were explained, and all participants signed an informed consent before participation in the study. After completion, the two health care professionals answered questions, blind for each other’s answers, about feasibility and face validity of the SVI-D in a digital questionnaire. These questions were:

- How long did it take you to administer the index? What do you feel about this duration?
- Do you have any comments on the formulation in questions? If yes, which questions does it concern and what should change in your opinion?
- Do you have any comments on the content or nature of the questions? If yes, which questions does it concern and what should change in your opinion?
- Do you miss any questions? If yes, which one(s)?
- Are there questions redundant in your opinion, if yes, which one(s)?
- This index is designed to measure social frailty, to what extent does it do so in your opinion?
- Do you think you will use this index in your organization? Can you explain?

## 3. Results

The results are discussed following the steps in the cross-cultural adaption process.

Steps 1–3

In the first step the original 40 items from the Social Vulnerability Index were translated from English into Dutch in two separate Dutch translations. Six items were translated differently by the two professionals. In the second step, these items were discussed and a synthesis of the two translations was made in a new concept-version of the index. In the third step, after the back translation was performed by a native speaker, the two professionals did not find any significant differences compared to the original version of the index.

Step 4 Expert committee: the Delphi procedure

Round 1

Of the 20 contacted experts, 13 responded by filling out the digital questionnaire in the first round, five experts did not respond, and two declined to participate due to various reasons.

In the first round, the 40 items were included in the index from the translation from the first three steps. The 13 experts made comments on the formulation of various items. Thirty-nine items of the index in the first round were scored “suitable” or “very suitable” by the panel, and only one item did not meet up to this 70% cut-off point. This item (“How often work in the garden”) was consequently removed from the SVI-D for the second round. No new items were added by the experts.

All experts had comments on the formulation of items in the first round concerning the formulation of nearly half of the total amount items. These comments were diverse and concerned for example the choice of specific words, the way questions were asked, or the availability of answers in multiple choice questions. Therefore, an extra step was implemented in the cross-cultural adaptation process: the initial translation of the remaining 39 items were revised by an expert in Dutch language, without knowledge of concepts and purpose of the index. However, this expert had a substantial background in writing for, and about, older adults in the Dutch language. This expert, therefore, has rewritten the items using the feedback of the experts. The purpose of this step was to process the feedback of the experts, in order to improve the initial Dutch translation in the whole index. This has led to an adjusted formulation of the remaining 39 items in the index.

Round 2

In the second round we approached the 13 experts that responded in the first round, as well as the five experts who had not initially responded. Ten experts, who also participated in the first round, filled out the questionnaire. Two experts declined continued participation due to various reasons (for example, a shortage of time). Six experts did not respond. Of the remaining 39 items, 33 items met the cut-off point of 70% agreement on the suitability of items for measuring social vulnerability in older adults, according to the expert panel. Six items did not meet this cut-off point. These items were then removed from the list of items for the third round. These items were “Number of people spend time with regularly”, “Feel need to spend more time with friends/family”, “People would describe me as a giving person”, “How do you feel about your life in terms of … finances, religion, transportation, and life in general” (four items), “Does income currently satisfy needs”, and “Home ownership”. The comments made by the experts on the formulation of the remaining items were all processed in the items in the prefinal version for the third round of the Delphi procedure: all suggestions for improvement of the formulation were accepted by the first author (SB).

Round 3

In the third round, the 10 experts who had completed the questionnaire in the second round, as well as the six experts who had not responded in the second round, were contacted to fill out the questionnaire in the third round. Seven experts filled out the questionnaire. Nine experts did not respond. The final result of third Delphi round was that the vast majority agreed on the final 32 items version of the SVI-D. One expert did not agree, with the main reason being that the variables “gender” and “age” were not part of the list of items. However, six experts agreed upon the final version of the 32 items SVI-D.

Step 5 Testing the prefinal version: face validity and feasibility

Two health care professionals, with similar professional backgrounds and experience, administered the 32 items SVI-D in two separate samples of 28 community-dwelling older adults. These older adults were already under treatment for rehabilitation, and were living independently. One professional reported an average time of administering the SVI-D of five minutes, and the other one reported about 30 min to administer. Both professionals reported just a few remarks concerning formulation of items concerning the use of some specific words. As for the nature and content of items, both professionals were satisfied and reported no specific remarks. All items were understandable for the interviewed older adults. One professional reported that there was no item referring to situations of acute vulnerability (when one needs instant help), and no item referring to life goals of the older adult being was interviewed. The other professional reported no items missing. Both professionals thought all items were relevant for measuring social vulnerability.

## 4. Discussion

The main result of this study is a face-valid and feasible 32 item Dutch version of the Social Vulnerability Index (Appendix B). The SVI-D is an index to quantify social vulnerability in Dutch-language countries, and offers a broad, holistic quantification of older people’s social circumstances, related to the risk of adverse health outcomes. Being able to identify social frailty in older adults is useful, both for designing interventions and policy as well as for the prediction of health outcomes [9,10].

The SVI-D includes a variety of items that measure aspects of all components of this conception of social frailty. Doing so, it enables a broad, holistic identification of aspects of social frailty. However, it remains unclear what the relative weight and importance of the different components of social frailty are in their effect on (un)fulfillment of (individual) people’s specific social needs. On the other hand, in using the SVI in prior epidemiological studies, weighting of individual items was found to be unnecessary, as no single item or clusters of items have been found to drive associations with health outcomes [9]. The amount of social factors and interactions that influence health is large, and therefore difficult to measure. This justifies the use of a broad and relatively extended index, as the SVI-D.

In adapting the index for the Dutch language and culture, the experts discarded several items from the original index. These items were not found sufficiently applicable to the Dutch context (for example, playing golf, which is at this moment only applicable to a small proportion of older adults in The Netherlands). Furthermore, no new items were added. This leads to the question whether there are indeed no specific items for the Dutch context which were not already in the initial index. It could be hypothesized that the items that were already present in the initial SVI were quite generic for populations worldwide. On the other hand, the concept of social frailty might be a relative new concept for the experts that were consulted. This suggests that it has been hard for them to define which items should be part of a Dutch SVI and which items should not. The two additional items suggested by one panel member (sex and age) are no doubt important for social vulnerability. However, because of their singular importance in health and social research, we argue that they should be considered separately to enable study of age and gender effects, for example in stratified analyses or as confounders or effect modifiers.

### Strengths and Limitations

A strength of this study is that we used a systematic method to adapt the SVI to the Dutch language and culture. It was of added value to integrate a Delphi-procedure in the steps of this method because of the scientific and professional expertise that was used in adapting the index. The expert panel was composed by experts in clinical medicine and social sciences, as well as by health care professionals. This offers a broad scientific and professional scope on the potential relevance of the included items in the SVI-D. In the cultural adaptation process, we implemented a Delphi method, which implies a variability in data, depending on the opinions of experts involved. Another expert panel might have given other results. However, the Delphi method is a well-known methodology where experts can give their opinion freely and anonymously [9].

The SVI-D is tested only in a pilot-sample of 28 community-dwelling elderly people by two health care professionals. In a next step, testing the scale in a larger sample is required to ensure the index’s psychometric properties. Moreover, the operationalization of social vulnerability as used in the SVI-D is based on self-report data rather than on objectively defined social factors. This might potentially cause some bias in the results, as it is known that self-reported data include over-reporting or under-reporting of the interviewed [15]. On the other hand, an older person’s subjective experience of social isolation or support may in itself contribute importantly to her/his overall level of vulnerability.

There was a high rate of expert drop-out in our study, which might have biased the results. Participant drop-out is one of the known methodological challenges in Delphi research [16]. One possible reasons for this high drop-out is that we contacted the experts via email, and not personally. It is known that personal contact in recruiting respondents increases participation. Another reason might be that experts in this study both had to look at a considerable number of items, both in terms of formulation as well as their suitability for the Dutch context. This may have taken too much of their time, and might have led to drop-out of some of the experts. In order to generate a comprehensive first draft of suitable items, it might have been better to have the first draft considered only by a small expert panel (of 2–4 experts) before sending it to a larger group. Doing so, this might have led to a more rigorous first draft of the index, and the experts in the broader panel could have been more specific in their feedback in the first round. Consequently, they might have needed less time answering the questionnaire because some issues then already had been addressed by the small expert panel.

As for the composition of the expert panel, the study design could have been strengthened by including older adults, or organizations that represent them, into the expert panel. In future studies, this could lead to other insights than those of scientific experts or health care or welfare professionals working with older adults.

With regard to the feasibility of the index, we saw a great difference in the time to administer the index. The professional who took half an hour to administer the index noted that the questions led to the participant to tell his or her “life story” and have a conversation about several answers. The other professional only took 5 min to administer the index. This suggests there is a variability in how professionals administer the index, for example if they invite the interviewed older adults to discuss their life situation in a more extended way. The fact that age as a factor influencing social vulnerability is not part of the index (as was the reason of one expert not to agree on the final version of the SVI-D) is because age is rather a risk factor of becoming socially frail, rather than being a component of social frailty as such [8].

## 5. Conclusions

The SVI-D is a useful index to measure social frailty in Dutch-language countries and offers a broad, holistic quantification of older people’s social circumstances, related to the risk of adverse health outcomes. This is, to our knowledge, the first index that aims to measure social frailty in a Dutch-language population. Being able to identify socially frail older adults, and their specific needs, is useful for designing interventions and policy, both on the level of individual older adults, as well as in the population.

## Appendix A. A 40-Item Version of the Social Vulnerability Index

**Communication to engage in wider community**

1 Read English or French

2 Write English or French

**Living situation**

3 Marital status

4 Lives alone

**Social support**

5 Someone to count on for help or support

6 Feel need more help or support

7 Someone to count on for transportation

8 Feel need more help with transportation

9 Someone to count on for help around the house

10 Feel need more help around the house

11 Someone to count on to listen

12 Feel need more people to talk with

13 Number of people spend time with regularly

14 Feel need to spend more time with friends/family

15 Someone to turn to for advice

16 Feel need more advice about important matters

**Socially oriented Activities of Daily Living**

17 Telephone use

18 Get to places out of walking distance

**Leisure activities**

19 How often visit friend or relatives

20 How often work in garden

21 How often golf of play other sports

22 How often go for a walk

23 How often go to clubs, church, community centre

24 How often play cards or other games

**Ryff scales**

25 Feel empowered, in control of life situation

26 Maintaining close relationships is difficult and frustrating

27 Experience of warm and trusting relationships

28 People would describe me as a giving person

**How do you feel about your life in terms of …**

29 Family relationships

30 Friendships

31 Housing

32 Finances

33 Neighbourhood

34 Activities

35 Religion

36 Transportation

37 Life generally

**Socio-economic status**

38 Does income currently satisfy needs

39 Home ownership

40 Education

## Appendix B. The Dutch Version of the Social Vulnerability Index, the SVI-D

**Table:**

Vraag	Antwoord	Score
1. Spreekt u The Nederlands?	☐ ja *(0 pnt)* ☐ nee *(1 pnt)* ☐ een beetje *(0.5 pnt)*	
2. Kunt u The Nederlands lezen?	☐ ja *(0 pnt)* ☐ nee *(1 pnt)* ☐ een beetje *(0.5 pnt)*	
3. Heeft u een partner? (maak een keuze)	☐ Ik woon samen met mijn partner (ga verder met vraag 5) *(0 pnt)* ☐ Ik heb een partner en we wonen ieder apart (ga verder met vraag 5) *(0 pnt)* ☐ Ik heb geen partner (ga verder met vraag 4) *(1 pnt)*	
4. Indien u geen partner heeft, hoe woont u? (maak een keuze)	☐ Ik woon zelfstandig in een vrijstaand huis *(1 pnt)* ☐ Ik woon zelfstandig in een serviceflat *(1 pnt)* ☐ Ik woon bij mijn kinderen *(0 pnt)* ☐ Ik woon samen met een vriend of vriendin *(0 pnt)* ☐ Ik woon in een woongroep of instelling *(0 pnt)* ☐ Ik heb geen vaste woon- of verblijfplaats *(1 pnt)* ☐ Bovenstaande opties zijn niet van toepassing *(0 pnt)*	
*Geef van de volgende stellingen aan of ze op u van toepassing zijn, of niet.*		
5. Als ik praktische hulp of steun nodig heb, is er iemand op wie ik kan rekenen.	☐ Ja *(0 pnt)* ☐ Soms *(0.5 pnt)* ☐ Nee *(1 pnt)*	
6. Ik heb behoefte aan meer praktische hulp of steun	☐ Ja *(1 pnt)* ☐ Soms *(0.5 pnt)* ☐ Nee *(0 pnt)*	
7. Als ik emotionele hulp of steun nodig heb, is er iemand op wie ik kan rekenen.	☐ Ja *(0 pnt)* ☐ Soms *(0.5 pnt)* ☐ Nee *(1 pnt)*	
8. Ik heb behoefte aan meer emotionele hulp of steun.	☐ Ja *(1 pnt)* ☐ Soms *(0.5 pnt)* ☐ Nee *(0 pnt)*	
9. Als ik ergens heen moet, is er iemand die me brengen kan.	☐ Ja *(0 pnt)* ☐ Soms *(0.5 pnt)* ☐ Nee *(1 pnt)*	
10. Ik heb vaker behoefte aan iemand die me ergens heen brengen kan.	☐ Ja *(1 pnt)* ☐ Soms *(0.5 pnt)* ☐ Nee *(0 pnt)*	
11. Als ik hulp nodig heb bij huishoudelijk werk, dan is er iemand op wie ik kan rekenen.	☐ Ja *(0 pnt)* ☐ Soms *(0.5 pnt)* ☐ Nee *(1 pnt)*	
12. Ik heb behoefte aan meer hulp bij het huishouden.	☐ Ja *(1 pnt)* ☐ Soms *(0.5 pnt)* ☐ Nee *(0 pnt)*	
13. Als ik behoefte heb aan gezelschap en even praten, is er iemand op wie ik kan rekenen.	☐ Ja *(0 pnt)* ☐ Soms *(0.5 pnt)* ☐ Nee *(1 pnt)*	
14. Ik zou graag wat meer mensen hebben om mee te kunnen praten.	☐ Ja *(1 pnt)* ☐ Soms *(0.5 pnt)* ☐ Nee *(0 pnt)*	
15. Als ik advies nodig heb over belangrijke zaken is er iemand bij wie ik terecht kan.	☐ Ja *(0 pnt)* ☐ Soms *(0.5 pnt)* ☐ Nee *(1 pnt)*	
16. Ik heb meer behoefte aan advies over belangrijke zaken.	☐ Ja *(1 pnt)* ☐ Soms *(0.5 pnt)* ☐ Nee *(0 pnt)*	
17. Ik gebruik de telefoon om contacten te onderhouden.	☐ Ja *(0 pnt)* ☐ Soms *(0.5 pnt)* ☐ Nee *(1 pnt)*	
18. Ik kan plaatsen die te ver zijn om te lopen toch bereiken.	☐ Ja *(0 pnt)* ☐ Soms *(0.5 pnt)* ☐ Nee *(1 pnt)*	
19. Ik bezoek vrienden en/of familie, of zij bezoeken mij.	☐ Vaak *(0 pnt)* ☐ Soms *(0.5 pnt)* ☐ Nooit *(1 pnt)*	
20. Ik fiets en/of wandel samen met anderen.	☐ Vaak *(0 pnt)* ☐ Soms *(0.5 pnt)* ☐ Nooit *(1 pnt)*	
21. Ik sport in groepsverband.	☐ Vaak *(0 pnt)* ☐ Soms *(0.5 pnt)* ☐ Nooit *(1 pnt)*	
22. Ik speel gezelschapsspellen.	☐ Vaak *(0 pnt)* ☐ Soms *(0.5 pnt)* ☐ Nooit *(1 pnt)*	
23. Ik ga naar een club/vereniging, de kerk, en/of het buurthuis.	☐ Vaak *(0 pnt)* ☐ Soms *(0.5 pnt)* ☐ Nooit *(1 pnt)*	
24. Ik heb voldoende regie om zelf te bepalen hoe ik leef.	☐ Ja *(0 pnt)* ☐ Nee *(1 pnt)*	
25. Ik vind het moeilijk om vriendschappen te onderhouden.	☐ Ja *(1 pnt)* ☐ Nee *(0 pnt)*	
26. Ik heb warme en fijne vriendschappen.	☐ Ja *(0 pnt)* ☐ Nee *(1 pnt)*	
27. Maak een keuze uit één van de drie stellingen:	☐ Ik word gelukkig/blij van de contacten met mijn familie in mijn leven *(0 pnt)* ☐ Ik word verdrietig/boos van contacten met mijn familie in mijn leven *(1 pnt)* ☐ Neutraal: geen van beide bovenstaande stellingen is van toepassing *(0.5 pnt)*	
28. Maak een keuze uit één van de drie stellingen:	☐ Ik word gelukkig/blij als ik denk aan de vriendschappen in mijn leven *(0 pnt.)* ☐ Ik word verdrietig/boos als ik denk aan de vriendschappen in mijn leven *(1 pnt)* ☐ Neutraal: geen van beide bovenstaande stellingen is van toepassing *(0.5 pnt)*	
29. Maak een keuze uit één van de drie stellingen:	☐ Ik ben tevreden/gelukkig met mijn woonsituatie *(0 pnt)* ☐ Ik ben ontevreden/ongelukkig met mijn woonsituatie *(1 pnt)* ☐ Neutraal: geen van beide bovenstaande stellingen is van toepassing *(0.5 pnt)*	
30. Maak een keuze uit één van de drie stellingen:	☐ Ik ben tevreden/gelukkig met de buurt waar ik woon *(0 pnt)* ☐ Ik ben ontevreden/ongelukkig met de buurt waar ik woon *(1 pnt)* ☐ Neutraal: geen van beide bovenstaande stellingen is van toepassing *(0 pnt)*	
31. Maak een keuze uit één van de drie stellingen:	☐ Ik word gelukkig/blij van de activiteiten die ik onderneem *(0 pnt)* ☐ Ik word ontevreden/ongelukkig met de activiteiten die ik onderneem *(1 pnt)* ☐ Neutraal: geen van beide bovenstaande stellingen is van toepassing *(0 pnt)*	
32. Wat is de hoogste opleiding die u hebt gedaan?	☐ Lagere school *(1 pnt)* ☐ Ambachtsschool/LTS *(1 pnt)* ☐ Huishoudschool *(1 pnt)* ☐ Mulo *(1 pnt)* ☐ HBS *(0.5 pnt)* ☐ MMS *(0.5 pnt)* ☐ Lyceum *(0.5 pnt)* ☐ Atheneum *(0.5 pnt)* ☐ Gymnasium *(0.5 pnt)* ☐ Lagere beroepsopleiding *(1 pnt)* ☐ Middelbare beroepsopleiding *(0.5 pnt)* ☐ Hogere beroepsopleiding *(0 pnt)* ☐ Universiteit *(0 pnt)* ☐ Anders, bijvoorbeeld een interne opleiding: ………. *(1 pnt)*	
Totale score (punten optellen)		

De totale score is een getal tussen 0 en 32. Hoe hoger de score, hoe hoger de mate van sociale kwetsbaarheid.
